# Spatiotemporal changes in Cx30 and Cx43 expression during neuronal differentiation of P19 EC and NT2/D1 cells

**DOI:** 10.1002/cbi3.10005

**Published:** 2013-08-30

**Authors:** Carthur K Wan, Simon J O'Carroll, Sue-Ling Kim, Colin R Green, Louise F B Nicholson

**Affiliations:** 1 Faculty of Medical and Health Sciences, Department of Anatomy with Radiology and Centre for Brain Research, The University of AucklandAuckland, 92019, New Zealand; 2 Faculty of Medical and Health Sciences, Department of Ophthalmology, The University of AucklandAuckland, 92019, New Zealand

**Keywords:** cell differentiation, intercellular communication, in vitro models

## Abstract

While connexins (Cxs) are thought to be involved in differentiation, their expression and role has yet to be fully elucidated. We investigated the temporal expression of Cx30, Cx36 and Cx43 in two in vitro models of neuronal differentiation: human NT2/D1 and murine P19 cells, and the spatial localisation of Cx30 and Cx43 in these models.

A temporal Cx43 downregulation was confirmed in both cell lines during RA-induced neuronal differentiation using RT-PCR (*P* < 0.05) preceding an increase in neuronal doublecortin protein. RT-PCR showed Cx36 was upregulated twofold in NT2/D1 cells (*P* < 0.05) and sixfold in P19 cells (*P* < 0.001) during neuronal differentiation. Cx30 exhibited a transient peak in expression midway through the timecourse of differentiation increasing threefold in NT2/D1 cells (*P* < 0.001) and eightfold in P19 cells (*P* < 0.01).

Qualitative immunocytochemistry was used to examine spatiotemporal patterns of Cx protein distribution alongside neuronal differentiation markers. The temporal immunolabelling pattern was similar to that seen using RT-PCR. Cx43 was observed intracellularly and on cell surfaces, while Cx30 was seen as puncta. Spatially Cx43 was seen on doublecortin-negative cells, which may indicate Cx43 downregulation is requisite for differentiation in these models. Conversely, Cx30 puncta were observed on doublecortin-positive and -negative cells in NT2/D1 cells and examination of the Cx30 peak showed puncta also localized to nestin-positive cells, with few puncta on MAP2-positive cells. In P19 cells Cx30 was localized on clusters of cells surrounded by MAP2- and doublecortin-positive processes. The expression pattern of Cx30 indicates a role in neuronal differentiation; the nature of that role warrants future investigation.

## Introduction

Gap junctions are responsible for cell-to-cell communication (Sohl et al., 2004) representing a major route of intercellular signaling during development (Bruzzone and Dermietzel, 2006). Connexins (Cxs) are a multigenic protein family comprising the subunits of gap junctions. Gap junction channel properties vary due to the diverse properties of Cx subtypes (Bruzzone et al., 1996). Furthermore, new evidence suggests channel-independent roles for Cxs (Jiang and Gu, 2005) and both channel-dependent and -independent mechanisms of action may be relevant to regulation of neuronal differentiation as Cxs are now known to play a role in differentiation of various other tissues (Gu et al., 2003; Li et al., 2006; Langlois et al., 2007).

In vitro models have proven to be essential tools for fundamental research and as models of neural development, embryonal carcinoma (EC) cell lines have been widely used. The human neural-committed teratocarcinoma (NT2/D1) cell line is well established as a model of neural development, undergoing differentiation into postmitotic neurons (Pleasure et al., 1992) and astrocytes (Bani-Yaghoub et al., 1999b) via treatment with the morphogen retinoic acid (RA) and mitotic inhibitors. The analogous murine P19 cell line has shown the capacity to differentiate into multiple lineages including neural lineages through biochemical induction (McBurney et al., 1982) and through RA treatment similarly undergoes neural differentiation into both neurons and glia (Jones-Villeneuve et al., 1982). Cx expression has been examined to varying degrees in these in vitro models, however, the spatial and temporal Cx profile remains incomplete.

Using a cell aggregation method to examine the P19 cell line, reduced expression of Cx43 and Cx26 was found between the immature precursor P19 cells and derivative neurons and glia (Belliveau et al., 1997). The new adherent protocol and timecourse of P19 differentiation used in our study (Monzo et al., 2012) differs from this earlier aggregation study, thus Cx43 expression has not yet been examined across the range of methods. Furthermore, there is a dearth of information on the expression of Cx30 during the timecourse of P19 differentiation.

Similarly, spatiotemporal patterns of Cx expression have yet to fully elucidated in the analogous NT2/D1 cells. Bani-Yaghoub et al. (1997) reported a 70% reduction in Cx43 mRNA expression after 1 week RA treatment, with a progressive downregulation of this subtype over a further 3 weeks RA treatment. Another study reported Cx expression using a 6-week RA treatment of NT2/D1 cells (Boucher and Bennett, 2003). They showed differential expression of Cx43 and Cx36 between undifferentiated NT2/D1 cells and mature derivative neuronal cultures, however, did not examine the transient spatiotemporal changes that may occur during the timecourse of differentiation. Furthermore, in this model there also remains a lack of knowledge of changes in Cx30 and Cx36 expression during neuronal differentiation.

The objective of the present study was to examine and compare the spatial and temporal expression of Cx30 and Cx43, during neuronal differentiation in both the human NT2/D1 and murine P19 in vitro models. The temporal expression of the Cx36 was also examined.

## Materials and methods

### Cell culture and differentiation

NT2/D1 cells were cultured in DMEM/F12 with 10% FBS and 2 mM l-glutamine. The NT2/D1 cell line was differentiated using a protocol modified from Pleasure et al. (1992). Cells were replated at a density of 3 × 10^4^ cells/cm^2^ in T75 flasks and supplemented with 10 µM RA. Cells were grown for 4 weeks in this differentiation medium before each flask was replated 1:1 into T175 flasks in normal growth medium and grown for 2 days. Differentiated cells were subsequently replated (4 × 10^5^ cells/cm^2^) in DMEM/F12 with 5% FBS, 2 mM l-glutamine and a mitotic inhibitor cocktail (1 µM AraC; 10 µM FUDR; 10 µM Urd, Sigma–Aldrich) for an additional 12 days.

P19 EC cells were cultured in α-MEM with 10% FBS and 2 mM l-glutamine. The P19 EC cells were differentiated using an adherent protocol recently reported (2012); cells were plated (6 × 10^4^ cells/cm^2^) in α-MEM with 2.5% FBS and 1 µM RA into T75 flasks. After 4 days in RA, cells were replated (9 × 10^4^ cells/cm^2^) and grown for 5 days in NBA with N2 supplement, followed by 5 days in NBA with B27 supplement, 2 mM l-glutamine and a mitotic inhibitor cocktail (8 µM AraC; 8 nM 2′-deoxycytidine, Sigma–Aldrich).

### RT-PCR

Total RNA was extracted from NT2/D1 and P19 EC cells using an Illustra Triple Prep kit (GE Healthcare) according to the manufacturer's protocol. For NT2/D1 cells RNA was extracted prior to RA treatment and at 7, 14, 21 and 28 days during RA treatment; and for P19 EC cells RNA was extracted prior to RA treatment and at 0, 2, 4, 6, 8 and 10 days post-RA treatment. Samples were collected from three independently grown replicates for each timepoint. cDNA was synthesized using SuperScript III reverse transcriptase (Life Technologies) following the standard protocol and using 1 µg of total RNA. PCR reactions were performed using gene specific primers (Table 1) and Platinum *Pfx* DNA polymerase (Life Technologies) (Table 2). PCR reactions were run on 2% agarose gels containing 1× SYBR Safe DNA gel stain using gel electrophoresis at 100 V constant. Bands were visualized using a LAS-3000 CCD imaging system and software suite (FujiFilm). Reverse transcription reactions were also run in the absence of the reverse transcriptase enzyme and PCR reactions were run in the absence of cDNA template to ensure that DNA contamination of the RNA samples and PCR reagents had not occurred.

**Table 1:** Primer sequences for PCR for human and mouse Cxs

Target gene	Primer sequence (5′–3′)		Product length (bp)
hCx30	Sense	GTG ACG AGC AAG AGG ACT TC	512
	Antisense	CAG CAG CAG GTA GCA CAA CT	
hCx36	Sense	AAC GCC GTC ACT CTA CAG TC	596
	Antisense	CCT TGG CAG GTC CTT GTT AC	
hCx43	Sense	TAC AGC CAC TAG CCA TTG TGG ACC	435
	Antisense	CCC CTC ATT CAC ATA CAC AGA ACC	
mCx30	Sense	TGT GGC CGA GTT GTG TTA CC	400
	Antisense	ACT CCA AGG CCC AGT TGT CA	
mCx36	Sense	GCA CCC CCA GTC TCT GTT TTA T	401
	Antisense	AGA AAG TAC TGG CCC ACC AGA A	
mCx43	Sense	CTG CCT TTC GCT GTA ACA CTC A	400
	Antisense	GCA CTC AGG CTG AAC CCA TAG	
β-actin	Sense	GCT CGT CGT CGA CAA CGG CTC	353
	Antisense	CAA ACA TGA TCT GGG TCA TCT TCT C		

**Table 2:** Thermal cycling parameters for PCR

Target gene	Denature	Anneal	Extend	Cycles
hCx30	94°C, 30 sec	50°C, 60 sec	68°C, 90 sec	30
hCx36	94°C, 30 sec	50°C, 60 sec	68°C, 90 sec	30
hCx43	94°C, 30 sec	56°C, 60 sec	68°C, 60 sec	30
mCx30	94°C, 30 sec	60°C, 60 sec	68°C, 90 sec	30
mCx36	94°C, 30 sec	60°C, 60 sec	68°C, 90 sec	30
mCx43	94°C, 30 sec	60°C, 60 sec	68°C, 90 sec	30
β-actin	94°C, 30 sec	56°C, 60 sec	68°C, 60 sec	30

### Semi-quantitative statistical analysis

Semi-quantitative analyses of DNA agarose gel images were performed using the MacBiophotonics ImageJ software. Using the gel analysis plug-in features the lanes containing bands at the predicted molecular weights were selected and the Plot Lanes function used to provide pixel value histograms for each band. The area under the peak of each histogram was taken as the integrated density measurement of band intensity. These values were corrected for sample loading by normalisation against the constitutively expressed gene product, beta-actin. Replicate values were subsequently normalized against the average integrated density of all the bands on each respective blot or gel to account for *per* experiment variation. Statistical analysis of expression values derived by this measure were compared using One-Way ANOVA, with *post-hoc* comparisons between timepoints performed using Tukey's test. The threshold for statistical significance was defined as *P* < 0.05. Data are presented as mean ± SE.

### Immunocytochemistry and confocal imaging

For immunocytochemistry (ICC), cells were grown in 96-well imaging plates (BD Biosciences) and fixed using 4% w/v PFA in 1 × PBS with Ca^2+^ and Mg^2+^ for 30 min at RT. Cells were washed with PBS and a blocking buffer (4% v/v NDS/NGS, 1% w/v BSA and 0.1% Triton-X 100 in PBS pH 7.4) was applied for 30 min at RT. The primary antibody was diluted in Ab solution (2% v/v NDS/NGS, 0.5% w/v BSA and 0.05% Triton-X 100 in PBS) at the concentrations indicated in Table 3 and applied to cells for incubation overnight at 4°C. After washes, secondary antibody was diluted in Ab solution and applied at room temperature for 1 h. Cells were subsequently washed and Hoescht applied before the addition of CitiFluor antifade reagent, which was then left to permeate at room temperature for 1 h prior to imaging. ICC was also performed in the absence of primary antibodies, to ensure there was no non-specific secondary antibody binding. Additionally, the primary antibodies for Cx30 and Cx43 have also been previously confirmed to be specific using cells induced to overexpress Cx30 and Cx43, respectively (Wan et al., 2012).

**Table 3:** Primary and secondary antibodies used for immunocytochemistry

Target protein	Species	Dilution	Manufacturer	Catalogue #
Primary antibodies				
Connexin 30	Rabbit	1:250	Zymed, Invitrogen	71–2200
Connexin 43	Rabbit	1:500	Sigma–Aldrich	C6129
Doublecortin	Goat	1:250	Santa Cruz	SC-8066
Nestin	Mouse	1:250	Chemicon	MAB5326
MAP2	Mouse	1:250	Chemicon	MAB3418
GFAP	Mouse	1:500	Sigma–Aldrich	G3893
Secondary antibodies				
AlexaFluor 488-conjugated Anti-Mouse IGG	Donkey	1:500	Invitrogen	A21202
AlexaFluor 488-conjugated Anti-Goat IGG	Donkey	1:500	Invitrogen	A11055
AlexaFluor 594-conjugated Anti-Rabbit IGG	Donkey	1:500	Invitrogen	A21207

ICC staining was imaged using a Zeiss LSM 710 inverted confocal microscope Plan-Apochromat 20×/0.8 NA dry objective (Carl Zeiss) and 63×/1.4 NA oil immersion objective and the ZEN 2009 software suite.

## Results

### Changes in Cx RNA expression during neuronal differentiation in the NT2/D1 and P19 EC cell culture models

**Figure 1:**
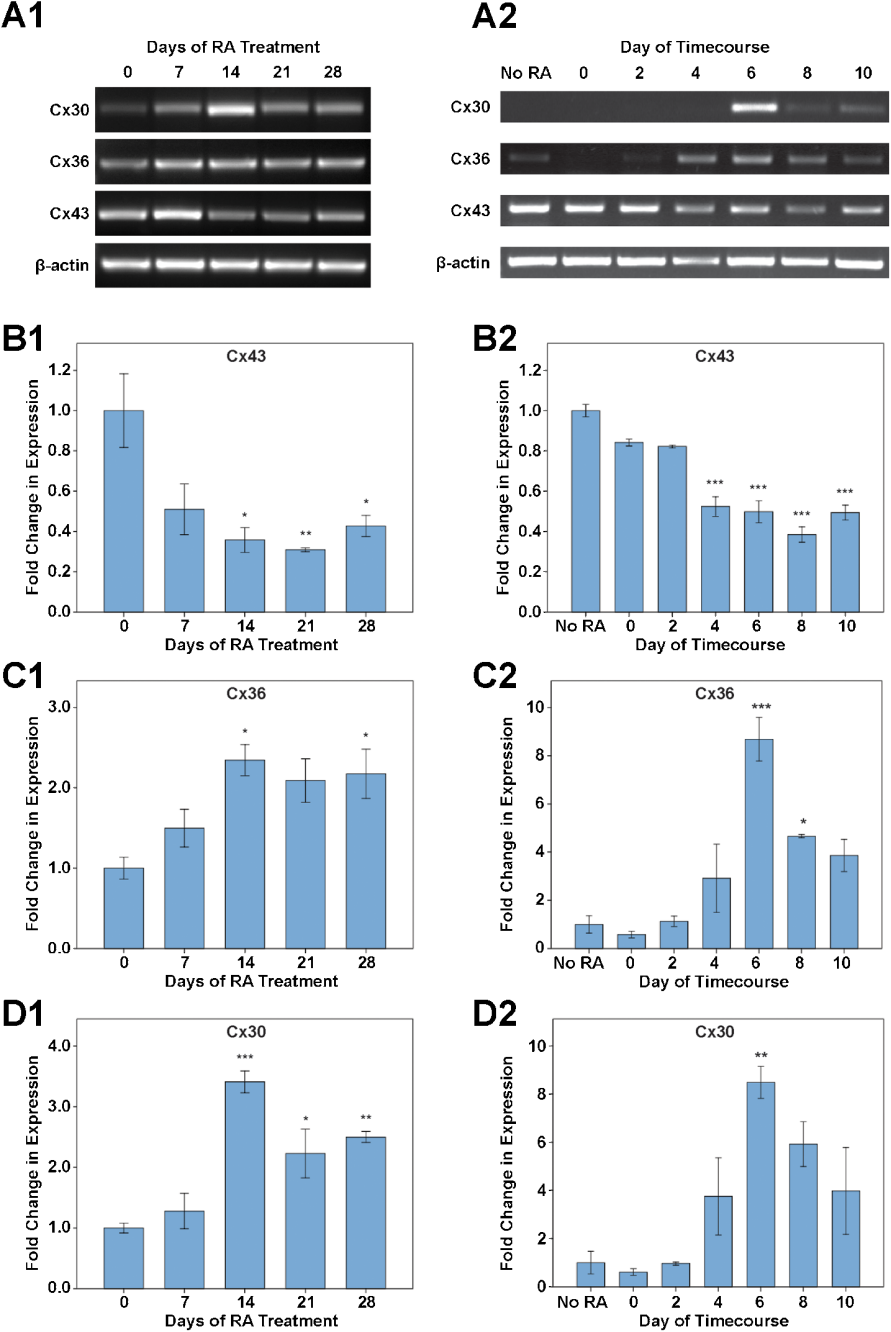
Temporal expression of Cx30, Cx36 and Cx43 mRNA during differentiation of the NT2/D1 and P19 EC cell models, as assessed by RT-PCR. Experiments were performed in triplicate independent cultures. Statistically significant changes relative to undifferentiated cells are denoted by asterisks (*P < 0.05; **P < 0.01; ***P < 0.001). (A) Representative images of the expression pattern of Cx30, Cx36 and Cx43 subtypes, and beta-actin, during the timecourse of NT2/D1 (A1) and P19 EC (A2) neuronal differentiation. A similar pattern of expression for the three Cx subtypes was seen in both cell lines. (B) Changes in Cx43 mRNA expression. In the NT2/D1 cell line, Cx43 decreased between day 0 and day 14 (*P* < 0.05) and remained downregulated at days 21 (*P* < 0.01) and 28 of RA treatment (*P* < 0.05) (B1). Cx43 significantly decreased in the P19 EC cell line by day 4 post-RA treatment and remained downregulated throughout the remainder of the timecourse (*P* < 0.001) (B2). (C) Changes in Cx36 mRNA expression. A significant increase in Cx36 expression was observed at 14 and 28 days RA treatment of NT2/D1 cells (*P* < 0.05) (C1), while in the P19 EC model a significant increase in Cx36 expression was observed at 6 (*P* < 0.001) and 8 days post-RA (*P* < 0.05) (C2). (D) Changes in Cx30 mRNA expression. In the NT2/D1 model, a threefold increase in expression of Cx30 was observed at the 14 days RA (*P* < 0.001), and the subtype was also elevated at days 21 (*P* < 0.05) and 28 (*P* < 0.01) (D1). A greater than ninefold increase in expression of Cx30 was observed at 6 days post-RA (*P* < 0.001) relative to undifferentiated P19 EC cells (D2).

The temporal expression patterns of Cx30, Cx36 and Cx43 mRNA were examined using RT-PCR, and semi-quantitative analysis was performed relative to the expression of the constitutive housekeeping gene, beta-actin, to provide a mean integrated density in arbitrary units (AU). The changes in Cx subtype expression levels were assessed relative to undifferentiated cells and the changes in expression level denoted in fold-change relative to undifferentiated cells (Figure 1A1 for NT2/D1 cells; Figure 1A2 for P19 EC cells).

In both the NT2/D1 and P19 EC cell lines Cx43 was downregulated early during differentiation. In the NT2/D1 cell line Cx43 mRNA levels showed an approximate threefold reduction from an initial expression level of 1.92 ± 0.35 AU within 14 days of RA treatment (*P* < 0.05, n = 3 independent cultures) and remained significantly downregulated throughout the remainder of the examined timecourse (Figure 1B1). In the P19 EC cell line Cx43 mRNA levels showed an approximate twofold decrease from an initial 1.53 ± 0.05 AU in untreated cells by 4 days post-RA treatment (*P* < 0.001, n = 3) and remained significantly downregulated at subsequent timepoints (Figure 1B2). Conversely, upregulation of both Cx30 and Cx36 subtypes was observed during differentiation in both cell lines.

In the NT2/D1 cell line Cx36 was significantly upregulated from an initial expression of 0.55 ± 0.08 AU in undifferentiated cells showing a greater than twofold increase by day 14 of RA (*P* < 0.05, n = 3; Figure 1C1). This twofold upregulation was sustained at day 28 of RA treatment (*P* < 0.05, n = 3). In the P19 EC cell line upregulation of Cx36 was more transient, peaking at day 6 post-RA treatment and showing an eightfold increase from a baseline of 0.31 ± 0.11 AU in undifferentiated cells (*P* < 0.001, n = 3; Figure 1C2). Cx36 remained significantly elevated at day 8 post-RA treatment (approximate fivefold increase; *P* < 0.05, n = 3). At day 10 post-RA treatment Cx36 expression was no longer significantly higher than in undifferentiated cells (*P* > 0.05, n = 3).

A transient increase in Cx30 mRNA expression was observed during NT2/D1 differentiation. Cx30 increased more than threefold by day 14 from a baseline expression level of 0.48 ± 0.03 AU (*P* < 0.001). Cx30 mRNA remained significantly elevated from undifferentiated NT2/D1 cells at later timepoints (day 21, *P* < 0.05; day 28, *P* < 0.01, n = 3; Figure 1D1). Similarly in the mouse P19 EC cell line Cx30 peaked transiently, showing a more than eightfold increase at day 6 post-RA treatment from an initial 0.28 ± 0.13 AU (*P* < 0.01, n = 3). Similarly to the NT2/D1 cells, in the P19 EC model Cx30 subsequently subsided and was no longer significantly elevated at day 10 post-RA treatment relative to undifferentiated cells (*P* > 0.05, n = 3; Figure 1D2).

No template and no reverse-transcriptase control reactions did not yield product.

### Spatiotemporal changes in Cx43 and Cx30 during neuronal differentiation of NT2/D1 and P19 EC cells

**Figure 2:**
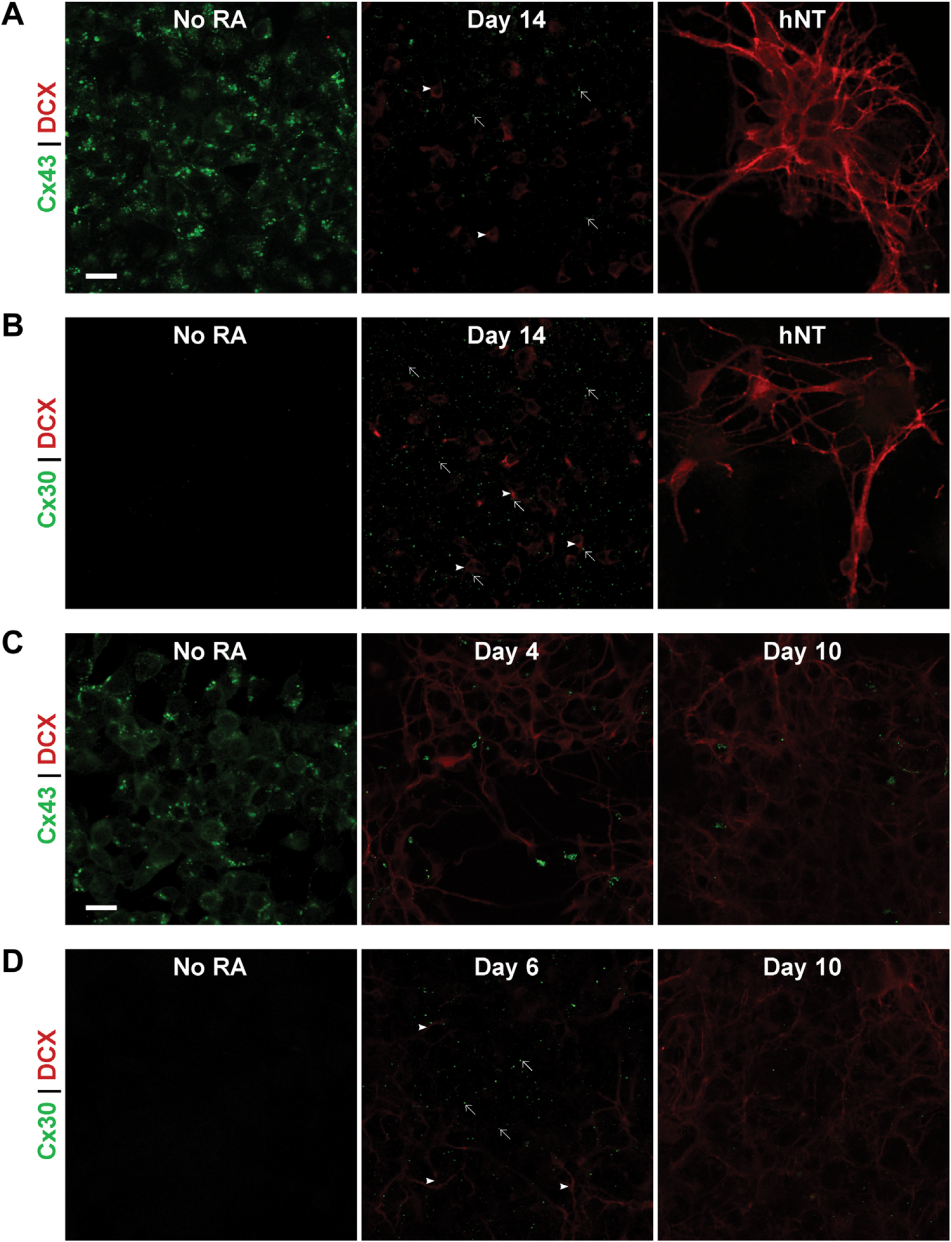
Spatial expression pattern of Cx43 and Cx30 protein with DCX as a marker of differentiating neurons in the NT2/D1 and P19 EC cell models. Scale bar = 20 µm. (A) Representative images of ICC labelling of Cx43 (green) and DCX (red) in undifferentiated NT2/D1 cells, cells at day 14 of RA treatment and mature hNT neuronal cultures. Intracellular and punctate labelling of Cx43 was observed in undifferentiated cells, however, immunolabelling for Cx43 was much less prevalent at day 14 (arrows) and appeared to largely be on cells without strong DCX staining rather than DCX-positive cells (arrowheads). In mature hNT neurons, Cx43 was not observed, while strong DCX was present in both cell bodies and neurites. (B) Representative images of ICC labelling of Cx30 (green) and DCX (red) in undifferentiated NT2/D1 cells, cells at day 14 of RA treatment and mature hNT neuronal cultures. Cx30 was not observed in undifferentiated NT2/D1 cells, however puncta became widespread peaking at day 14 of RA treatment, before decreasing again. Cx30 was not observed in cultures of hNT neurons. Cx30 puncta (arrows) were visible on DCX-positive cells (arrowheads) and on cells absent of DCX staining at day 14, with no obvious preferential localisation. (C) Representative images of ICC labelling of Cx43 (green) and DCX (red) in undifferentiated P19 EC cells, cells at day 4 post-RA treatment and day 10 post-RA treatment. Similar to NT2/D1 cells, Cx43 was observed as both intracellular and punctate labelling. Cx43 was initially ubiquitously expressed, however, both punctate and intracellular labelling decreased after RA treatment by day 4 post-RA treatment and remained low at day 10 post-RA treatment. (D) Representative images of ICC labelling of Cx30 (green) and DCX (red) in undifferentiated P19 EC cells, cells at day 6 post-RA treatment and day 10 post-RA treatment. Cx30 was not observed in undifferentiated P19 cells. At the 6-day timepoint post-RA treatment, Cx30 puncta were observed (arrows) at the centre of large clusters of cells with DCX-positive neurites (arrowheads) extending from them at their periphery. This peak in expression was transient as Cx30 decreased between day 6 and day 10 post-RA treatment.

ICC was used to examine the spatiotemporal pattern of Cx43 and Cx30 protein expression in NT2/D1 cells during the timecourse of neuronal differentiation (Figures 2A and 2B). Cx43 immunolabelling was observed as punctate staining between cells along apposed cellular membranes indicative of gap junction plaques, but globular labelling was also observed within the cytoplasm of cell bodies (Figure 2A). Intracellular labelling was observed predominantly in cultures of undifferentiated cells, prior to any treatment with RA, and at earlier timepoints during the timecourse, where localisation of labelling was largely perinuclear.

Cx43 immunolabelling decreased in a pattern similar to the temporal changes observed at the RNA level, with a reduction of both punctate and intracellular globular labelling observed. The intracellular Cx43 appeared to decrease rapidly during RA treatment and was not observed by day 3 of RA treatment. Punctate Cx43 labelling remained prevalent at 3 days of RA treatment but began to decrease between 5 and 7 days of RA treatment. Immunolabelling was sparse by day 14 and remained negligibly low at the later 21 and 28 day timepoints. The postmitotic neurons and underlying feeder cells in the hNT cell cultures also lacked strong Cx43 immunolabelling. Examination of double-labelling of Cx43 and DCX showed the downregulation of Cx43 appeared to precede the emergence of an abundance of DCX-positive differentiating neurons, and as such, colocalisation was negligible over the timecourse of differentiation.

The localisation of Cx30 during NT2/D1 differentiation was also investigated using ICC and double-labelled against the DCX neuronal marker (Figure 2B). When expressed, Cx30 was observed as punctate immunolabelling localised at cell-cell interfaces, giving rise to a honeycomb pattern of puncta, typical of gap junction formation. In contrast to the Cx43 subtype, Cx30 was predominantly observed as punctate labelling at the timepoints examined during the course of NT2/D1 differentiation.

The spatiotemporal pattern of Cx30 protein exhibited by ICC bore resemblance to the temporal changes in RNA expression observed via RT-PCR. Cx30 immunolabelling was not apparent in undifferentiated NT2/D1 cells. Labelling remained low at 3 days of RA treatment although some sparse punctate staining was apparent at this timepoint. An increase in Cx30 puncta was seen at day 5 of RA treatment. This increase continued with immunolabelling becoming widespread and peaking between 7 and 14 days of differentiation. Subsequently, after day 14 of RA treatment, Cx30 immunolabelling decreased and was not observed in hNT cultures.

The emergence of DCX immunolabelling at ∼7 days of RA treatment coincided temporally with the increase in Cx30 immunolabelling observed between 5 and 14 days of RA treatment. At the peak of Cx30 labelling, puncta were observed on the cell borders of DCX-positive cells, but were also seen amidst the cell population absent of DCX staining, indicating this Cx subtype was forming junctions between cells committing to both neuronal and non-neuronal fates with no clear preference (Figure 2B). It was, however, noted that Cx30 appeared generally to be present in areas of higher cell density in these heterogeneous cultures.

A similar pattern of expression as that observed in the NT2/D1 model was seen during differentiation of the murine P19 EC cells (Figures 2C and 2D). As with NT2/D1 cells, Cx43 immunolabelling was observed to localize both intracellularly and at apposed cellular surfaces, indicating the formation of gap junction plaques. Extensive and relatively ubiquitous Cx43 immunolabelling was observed prior to RA treatment, and at the day 0 timepoint post-RA treatment both globular and punctate Cx43 immunolabelling remained widespread (Figure 2C). Subsequently, a decrease in Cx43 staining occurred in a similar pattern to that seen through RT-PCR. Between 2 and 4 days post-RA treatment both intracellular Cx43 immunolabelling and punctate Cx43 labelling between cells began to decline and continued to diminish throughout the later timepoints investigated.

Examination of double-labelling showed this decline in Cx43 immunolabelling coincided with an increase in DCX immunolabelling. At the 0 day and 2 day post-RA timepoints, where Cx43 immunolabelling was sustained it was principally expressed on cells that had yet to express any DCX at detectable levels. Immunolabelling at the day 4 timepoint was observed on some DCX-positive cells interspersed amongst DCX-positive neuritic processes, but Cx43 appeared to be primarily localized to cells that were absent of DCX expression.

Where Cx30 immunolabelling was observed in P19 EC cells, it appeared predominantly as discrete puncta, giving the impression of a tessellated pattern (Figure 2D). Cx30 immunolabelling was not initially present in cultures of undifferentiated P19 EC cells, however, changes in Cx30 immunolabelling were observed during differentiation and appeared to follow, rather than precede, the expression of DCX in the P19 EC cell line. Cx30 puncta were first apparent at 4 days post-RA with the number of puncta observed increasing and peaking at day 6 post-RA. Some Cx30 immunolabelling was still present at days 8 and 10 post-RA treatment, but the number of puncta had diminished from the peak observed at day 6.

Cx30 puncta, when observed, were found to be localized predominantly within the centres of large, dense clusters of cells, suggesting that this subtype was selectively localized to the cell type(s) forming these clusters. These cell clusters, while displaying modest DCX labelling at their centres, exhibited DCX labelling in neuritic processes at their peripheries.

### Cell type localisation of Cx30 at the peak timepoint of expression during neuronal differentiation of NT2/D1 and P19 EC cells

The transient elevation in Cx30 expression was further examined by co-immunolabelling with the neuronal precursor marker nestin and the mature postmitotic neuronal market MAP2, as well as the astrocytic marker GFAP, in both the cell models at the peak timepoints of expression: day 14 of RA treatment for the NT2/D1 cells and day 6 post-RA treatment for the P19 EC cells. GFAP was negligible in both models at the timepoints examined.

**Figure 3:**
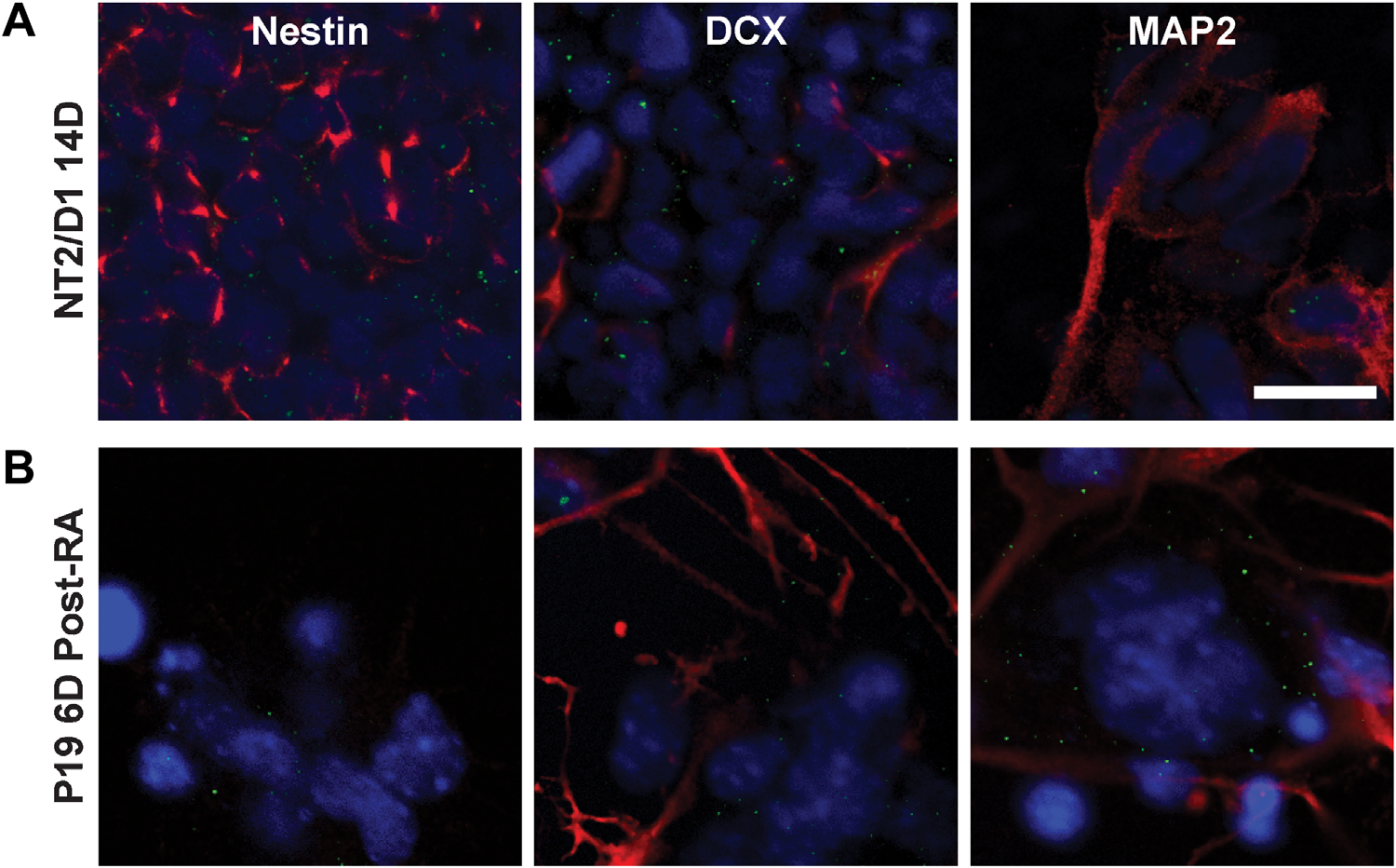
Cell type localisation of Cx30 at the peak timepoint of expression during differentiation of NT2/D1 and P19 EC cells. Scale bar = 20 µm. (A) Representative images of Cx30 (green), Hoescht (blue) and cell marker (red) double-labelling in the NT2/D1 cell line at day 14 of RA treatment. Nestin-positive cells formed a dense underlying layer. Interspersed amongst this layer of cells were DCX-positive differentiating neurons. Cx30 was predominantly seen as punctate labelling forming a cobblestone pattern around nestin- and DCX-positive cells in this underlying layer. A more superficial layer of MAP2-positive postmitotic neurons was also observed, with few sparse Cx30 puncta. (B) Representative images of Cx30 (green), Hoescht (blue) and cell-type marker (red) double-labelling in P19 EC cell line at 6 days post-RA treatment. Unlike the NT2/D1 cells, strong nestin immunolabelling was not observed, however, DCX-positive and MAP2-positive neurons were present. Cx30 puncta appeared to predominantly be expressed on clusters of underlying cells surrounded by DCX- and MAP2-positive cell bodies and neuritic extensions.

In the NT2/D1 model, nestin-positive cells were observed in abundance at day 14 forming a dense underlying layer of cells. Punctate Cx30 staining was localised in a cobblestone pattern at the borders of these nestin-positive cells (Figure 3A). Conversely, MAP2-positive cells were found in clusters of cells in a more superficial layer. DCX-positive cells were observed both superficially and interspersed with the underlying layer of cells. Cx30 puncta were observed predominantly on DCX-positive cells within the underlying layer. Sparse puncta were also observed in the superficial MAP2-positive cell layer.

In the P19 EC model, at day 6 post-RA treatment, DCX and MAP2 expression were observed in cells of neuronal morphology both somatically and dendritically (Figure 3B). Additionally, clusters of morphologically large flat cells were present that did not exhibit either of these neuronal marker. These cells may have been a P19 EC derived feeder layer and appeared to express a very low level of nestin. Cx30 appeared to be localized predominantly to these flat cell clusters, as well as to smaller cell bodies and neurites surrounding these cells, which were DCX-positive and MAP2-positive.

## Discussion

There is increasing evidence that Cxs serve as a regulatory link between various factors that impact neural lineage specification and differentiation. Changes in Cx subtype expression can affect the machinery governing cell cycle exit (Kamei et al., 2003; Zhang et al., 2003), alter gene expression (Iacobas et al., 2004) and modulate the extracellular microenvironment in the vicinity of neural precursors (Stout et al., 2002). However, the endogenous changes in Cx expression during neuronal differentiation have yet to be fully explored. Therefore, we have examined and compared the endogenous expression of three Cx subtypes in two in vitro models of neuronal differentiation in order to extend past observations.

The temporal pattern of Cx30, Cx36 and Cx43 expression were similar in both in vitro models used in our study, implying that the changes were species independent. This consistency in the spatiotemporal pattern between the two models may also suggest the specific pattern of expression was requisite, rather than incidental, to the differentiation process. Further evidence corroborating that these changes are substantive, rather than model specific, can be seen in the similarities between the changes observed in this in vitro study and in vivo findings in past studies using different models (Rozental et al., 2000; Leung et al., 2002; Arumugam et al., 2005).

Gross examination of temporal changes in Cx mRNA expression showed an increase in Cx36 during the timecourse of NT2/D1 and P19 EC differentiation. In our study we have shown that Cx36 mRNA upregulation occurs relatively early during differentiation, implying the Cx36 isoform is not only expressed in mature neurons, but in cells undergoing the process of neural differentiation in these in vitro models. The early upregulation of Cx36 in both models examined in this study poses the question as to whether the increase in expression observed is simply secondary to the development of neuronally-committed cells in these cultures, or whether this expression may, in fact, be a key feature in the commitment and differentiation of these cells to a neuronal fate. A recent study supports the latter, wherein it was reported that expression of the Cx36 gene was necessary for neuronal specification in primary cultures of hippocampal NPCs and mouse neurospheres (Hartfield et al., 2011). Cx36 may thus be serving a similar role in these in vitro models investigated in the present study with the temporal expression pattern observed reflecting that role.

As the second Cx subtype to have been identified, and perhaps the most ubiquitous of the protein family, Cx43 has been the most comprehensively studied Cx subtype to date. As such, previous examinations of Cx43 changes have been undertaken in the in vitro models used in our study, albeit with differences in the exact methodology used to culture both cell lines (Bani-Yaghoub et al., 1997; Belliveau et al., 1997; Boucher and Bennett, 2003). Our study has further corroborated the findings of those earlier studies investigating the expression of Cx43, in both the P19 EC and NT2/D1 models. Expression of Cx43 was highest in both the undifferentiated NT2/D1 and P19 EC precursor cells, with relatively ubiquitous expression in cultures of these cells and a marked decrease in this gap junction subtype occurred upon RA treatment.

A preponderance of Cx43 protein was observed both at the plasma membrane and intracellularly in both undifferentiated NT2/D1 and P19 EC cells. This cytoplasmic expression may simply result from the sheer magnitude of Cx43 expression, saturating the cellular machinery for translation and trafficking; the localisation within the cells appeared perinuclear and thus may correspond to the Golgi body and ER where Cx proteins are processed (Martin and Evans, 2004). The high degree of expression may also be indicative of the mitotic activity still occurring at these early timepoints with high levels of Cx43 expressed to enable the quick re-establishment of communication after cell division (Boassa et al., 2010). There is evidence to suggest a more causal relationship between the reduction in Cx43 and the progression of differentiation. This has been noted previously, for example in limb development where RA-induced reduction in Cx43 correlated with halted outgrowth (Green et al., 1994), and in skin wound healing where knockdown of Cx43 levels with specific antisense oligodeoxynucleotides changes cells from a proliferative to migratory phenotype (Qiu et al., 2003).

The downregulation of Cx43 was observed to largely precede the expression of the DCX protein, suggesting that a reduction of Cx43 was a requisite for neuronal differentiation in these in vitro models. There are differing opinions reported in the literature on the necessity of Cx43 GJIC during differentiation. It has been shown that gross pharmacological blockade of GJIC can impair differentiation in these in vitro models (Bani-Yaghoub et al., 1999a,1999c), leading to the suggestion that functional Cx43 may be required for neural differentiation. The agents used in such studies, however, are non-selective and would impact GJIC not only via the Cx43 subtype, but also other subtypes investigated in our study: Cx30 and Cx36. Conversely, it has also been shown that Cx43 expression and GJIC may instead be key to maintenance of a proliferative state (Cheng et al., 2004; Todorova et al., 2008). Therefore, this observed rapid downregulation of Cx43 may reflect a necessary reduction in GJIC to allow the NPCs to undergo cell cycle exit.

Further evidence that the observed reduction in Cx43 may be required for neuronal differentiation comes from Cx43 overexpression studies. Upregulation of Cx43 was shown to diminish the number of neuronal clusters produced from the NT2/D1 NPCs and reduce neurite outgrowth (Dahm et al., 2010). They did not, however, examine the effect on any other Cx subtypes, concurrent with this drug treatment, that may be influencing cell fate. Furthermore, aberrant Cx43 upregulation is a notable feature in acute brain injury (Ohsumi et al., 2006; Cronin et al., 2008; O'Carroll et al., 2008) and chronic neurological disorders (Fonseca et al., 2002; Collignon et al., 2006) in which preferential astrogliosis and/or depressed neuronal differentiation have been reported (Tatsumi et al., 2008; Hattiangady and Shetty, 2010).

Cx30 has previously been described as an astrocytic Cx subtype (Nagy et al., 1999; Rash et al., 2001). In this study, however, we have observed upregulation of Cx30 during RA-induced neuronal differentiation. NT2/D1 cells do not express astroglial markers such as GFAP and glutamine synthase and only undergo astrocytic differentiation in succession of the development of hNT neurons through the continued cultivation of the non-neuronal feeder layer (Bani-Yaghoub et al., 1999b; Sandhu et al., 2002). Similarly, we did not observe GFAP at the peak timepoint of Cx30 expression in the P19 EC model of differentiation.

The temporal expression of Cx30 in both models examined in this study, in fact, appeared to correspond to the temporal increase in DCX protein expression observed via ICC, in a fashion similar to the expression of the neuronal Cx36 gap junction, rather than the astrocytic Cx43. This would therefore suggest that the upregulation of Cx30 was occurring in either differentiating neurons, precursors of the eventual supporting non-neuronal feeder layer of cells or a combination of these two cell types rather than being due to mature astrocytic expression.

The upregulation of Cx30 appeared to be highly transient, peaking strongly before subsiding. This previously unreported elevation of expression reflects a limitation of earlier studies in which only differential expression between undifferentiated and mature cells was examined. ICC staining revealed a similar temporal pattern of Cx30 expression at the protein level, however, in the NT2/D1 cell line the spatial pattern of expression did not appear indicative of any clear preference for either the developing DCX-positive neurons or non-neuronal cells that can serve as precursors to astrocytes. Examination of Cx30 at day 14 alongside nestin and MAP2 indicated that Cx30 puncta were largely localized to a nestin-positive cell layer, while they were associated to a lesser degree with MAP2-positive postmitotic neurons. It may be that while Cx30 was necessary in the transition of cells from nestin-positive neuronal precursors to DCX-positive migratory neuroblasts, it was not required in postmitotic mature neurons.

Conversely, while the temporal pattern of expression in the differentiating P19 EC cells appeared similar to that of the NT2/D1 cells, the spatial pattern differed. Cx30 puncta were found largely in areas encompassed by DCX-positive and MAP2-positive cell bodies and neurites. This may suggest that in this mouse model of differentiation Cx30 was predominantly expressed by the developing neurons that extended these neurites, or alternatively, this expression may be occurring on surrounding and underlying cells supporting the development of these neurons. It may be that intermittent expression of Cx30 protein facilitates communication in these mixed cultures both homotypically between cells undergoing neuronal differentiation, and heterotypically between these cells and the surrounding non-neuronal cells that may promote and support differentiation (Toeg et al., 2007). Furthermore, it has been shown that neurons can induce the expression of astrocytic Cx30, thus, it is possible the developing neurons may be driving an upregulation of Cx30 in the non-neuronal cells in differentiating NT2/D1 and P19 EC cultures (Koulakoff et al., 2008).

Cx30 may, ultimately, simply be providing intermediary gap junctional communication in both models during a period in which Cx43 is downregulated and Cx36 upregulated. In such a manner, Cx30 upregulation may create a “communication compartment” between these developing neurons and supportive cells, while overall GJIC is becoming restricted. Cx30 is highly cation-selective and forms gap junction channels that are amongst the narrowest, while Cx43 is relatively unselective with regard to charge and forms relatively wide pores (Harris and Locke, 2008). The inverse regulation of Cx30/Cx36 and Cx43 may consequently impose greater selectivity or modify the electrochemical nature of GJIC between these cells.

A tightly regulated pattern of Cx expression was observed and of particular interest, is the previously unreported and transient elevation of the Cx30 subtype. Further studies are, however, needed to elucidate the functional implications of these changes in expression. A greater knowledge of endogenous Cx expression during differentiation will improve our understanding of their role during conditions of impaired neurogenesis and, consequently, modulation of their expression may ultimately serve as a basis for the development of therapeutic interventions.

## Abbreviations

α-MEM: minimal essential medium alpha
AraC: cytosine arabinoside
Cx: connexin
DCX: doublecortin
DMEM/F12: Dulbecco's modified Eagle medium with F12 supplement
EC: embryonal carcinoma
FBS: fetal bovine serum
FUDR: fluorodeoxyuridine
GJIC: gap junction intercellular communication
NBA: neurobasal-A
NT2/D1: neural-committed teratocarcinoma subclone D1
PBS: phosphate-buffered saline
RA: all-*trans* retinoic acid
TBS: tris-buffered saline
Urd: uridine
